# Identification of wheat proteins involved in active stage of celiac disease: are gamma gliadins the major disease-specific antigens?

**DOI:** 10.1186/2045-7022-1-S1-O22

**Published:** 2011-08-12

**Authors:** Bharani Srinivasan, Claudia Constantin, Margit Focke Tejkl, Innes Swoboda, Irene Mittermann, Harald Vogelsang, Wolf Dietrich Huber, Rudolf Valenta

**Affiliations:** 1 Division of Immunopathology, Department of Pathophysiology and Allergy Research, CePII, Medical University of Vienna, Vienna, Austria; 2 Division of Immunopathology, Department of Pathophysiology and Allergy Research, CePII, Christian Doppler Laboratory for Allergy Research, Medical University of Vienna, Vienna, Austria; 3 Department of Pediatrics and Adolescent Medicine, Medical University of Vienna, Vienna, Austria; 4 Department of Gastroenterology and Hepatology, Medical University of Vienna, Vienna, Austria

## Background

Celiac disease (CD) is caused by a severe immune response to wheat gliadins and glutenins. It is thought that gliadin-specific T cells mediate mucosal damage and generation of IgA/IgG class anti-gliadin antibodies, but individual protein antigens and epitopes have not been studied in detail.

## Objective

To characterize wheat antigens with ability to initiate and sustain CD.

## Methods

We developed a method wherein the alcohol extracted gliadins was fractionated in two steps of ion-exchange chromatography, Sulphopropyl (SP) was used for the first step and the flow through (FT) fraction obtained was further fractionated using DEAE. Each generated fraction’s reactivity to serum IgA, from clinically well defined CD (active/diet) patients and non-CD patients was analyzed. Identification of disease specific antigens in the fractions was attempted using mass spectrometry and N-terminal sequencing.

## Results

Patients with active disease showed strong IgA reactivity to proteins in all the fractions. Interestingly, we found that non-CD and CD patients under diet, exhibited background IgA reactivity which were mainly restricted to the elution fraction of SP but did not react with FT SP and FT DEAE fractions. The latter fractions hence appeared useful to identify patients with active forms of CD. Mass Spectrometry and N-terminal sequencing revealed that gamma-gliadins were enriched in FT SP and FT DEAE fractions.

## Conclusions

We report here a purification protocol for enriching IgA-reactive antigens specifically recognised by active CD patients. This fraction containing majorly gamma-gliadins will be useful for characterizing individual proteins, involved in disease and for developing diagnostic and treatment strategies.

